# Fibrous dysplasia of the head and neck in Southern Finland: a retrospective study on clinical characteristics, diagnostics, and treatment

**DOI:** 10.1007/s00405-024-08595-z

**Published:** 2024-04-02

**Authors:** Isabella Vilos, Mikko T. Nieminen, Riikka E. Mäkitie

**Affiliations:** 1 Department of Otorhinolaryngology, Head and Neck Surgery, Helsinki University Hospital and University of Helsinki, HUS, Kasarmikatu 11–13, 00029 Helsinki, Finland; 2 Faculty of Medicine, University of Helsinki, Helsinki, Finland

**Keywords:** Fibrous dysplasia, Head and neck, Bone lesion, Bisphosphonates, Surgery

## Abstract

**Purpose:**

Fibrous dysplasia (FD) is a rare genetic disease with benign bone tumors. FD can affect one (monostotic FD) or multiple bones (polyostotic FD), with craniofacial lesions being common. Because of its rarity, there are only few clinical reports on FD in the head and neck region and its clinical characteristics remain incompletely defined. This study aimed to determine patient demographics, symptoms, diagnostics, and given treatment in patients with FD of the head and neck in a Finnish population.

**Methods:**

A retrospective review on all patients diagnosed with or treated for FD of the head and neck at the Helsinki University Hospital during 2005–2020.

**Results:**

In total 74 patients were identified; 54% were male and the mean age 45 years. Overall 95% had monostotic FD. Mandibula and maxilla were the most common anatomic sites. Majority of patients had symptoms, most commonly pain and lesion growth, and 49% had extra-skeletal symptoms. For all, diagnosis was primarily based on imaging findings, biopsies were obtained from 41%. Altogether 54 patients (73%) were managed by observation only, 20 patients (27%) received treatment; ten bisphosphonates, six surgery and four both.

**Conclusion:**

Although highly variable in its clinical manifestations, head and neck FD lesions are often symptomatic and impose risk for extra-skeletal complications. Treatment is often conservative but should be individually tailored. Future studies are encouraged to better define the disease characteristics and hopefully offer new treatment possibilities.

## Introduction

Fibrous dysplasia (FD) is a rare genetic disease characterized by benign tumors in one (monostotic FD; MFD) or multiple bones (polyostotic FD; PFD), most typically in long bones, ribs, cranial bones, and the pelvis [1]. MFD is milder and more common, accounting for up to 70% of cases [2]. PFD is less frequent and occasionally linked to a syndrome, such as McCune-Albright syndrome (MAS) with endocrinopathies and café-au-lait spots [3], Mazabraud syndrome with skeletal muscle myxomas [4], or Jaffe-Lichtenstein syndrome with café-au-lait spots in the midline [1, 5]. FD is caused by a postzygotic activating missense-mutation in the *GNAS* gene [6] and the clinical presentation depends on the stage of embryonic development at which the mutation occurs, and on the distribution of mutated cells in the body [7]. In FD lesions, abnormal production of bone matrix, trabeculae, and collagen result in replacement of normal bone with connective tissue [8]. FD lesions typically develop in early childhood, and the majority of bone lesions and associated symptoms present during the first decade of life [9].

Due to the detailed and complex anatomy of the head and neck region, lesions affecting craniofacial structures can lead to devastating consequences. Although the most common symptom is reportedly a painless, slowly growing swollen lump [3], lesions can grow large and cause facial asymmetry and deformities, compression of the optic canal and vision impairment, or affect the ear canal or the ossicular chain leading to conductive hearing loss [10], to name a few. Diagnostics are based on clinical, radiological, histopathological, and genetic information. Treatment is tailored according to an individual's clinical findings and disease progression by a multi-disciplinary team and consists of surgery, medical treatment, and rehabilitation.

Given its rarity, clinical reports on FD of the head and neck are sparse and its clinical characteristics still incompletely defined. While accurate estimates on the prevalence of FD are difficult to make, earlier reports state FD to cause 5–7% of benign bone tumors [1]; in Finland approximately 10–14 cases per year [11]. We therefore set out to retrospectively examine patients with FD of the head and neck in a Finnish population to determine patient demographics, symptoms, diagnostics, and given treatment.

## Materials and methods

In this retrospective register study, we identified all patients diagnosed with or treated for FD in the head and neck region at the Helsinki University Hospital during 2005–2020. The search was based on medical charts assigned under the ICD-codes K10.83 and M85.0. We reviewed all patient records for age, sex, time of diagnosis, classification of disease (MFD, PFD or MAS), lesion location, and symptoms such as fractures, extra-skeletal symptoms, neurologic symptoms, and chronic pain. We collected data on radiologic findings and results from histopathological and genetic analyses if available. Furthermore, we collected data on treatment, including its indications, effects, and complications, as well as disease progression during follow-up. Microsoft Excel was used for data collection, analysis and illustrations. Z-test and Mann–Whitney U test were used for statistical comparisons; a *p* value less than 0.05 was considered statistically significant. Research permission for this study was granted by HUS Head and Neck Center (No. 33/2021).

## Results

### Patient characteristics and clinical presentation

In total 74 patients were identified, of which 34 (46%) were female and 40 (54%) male (*p* = 0.33). Mean age was 45.1 years (range 13–87 years); females were slightly older (48.2 years vs 42.5 years, *p* = 0.75). The cohort was primarily of Caucasian ethnicity.

**Fig. 1:**
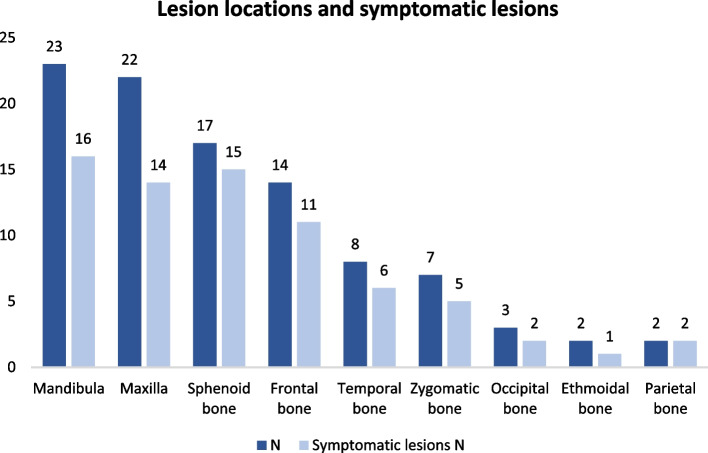
Distribution of lesions and symptoms in relation to anatomic site in 74 Finnish patients with head and neck fibrous dysplasia. Data show mandibula and maxilla as the most common lesion sites, while lesions in the sphenoid and parietal bones were most symptomatic

**Fig. 2:**
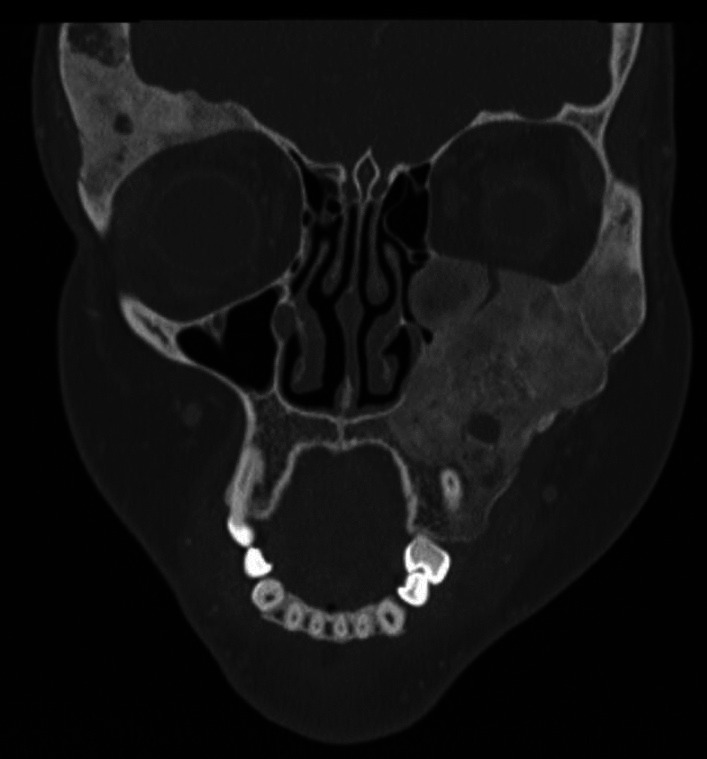
A coronal CT slice of paranasal sinuses of a 19-year-old female patient with polyostotic FD. Image shows lesions involving the left maxilla, mandibula, hard palate, zygomatic bone, temporal bone, left and right sphenoid bones, as well as the right frontal bone and the occipital bone. Lesions exhibit a typical ground-glass appearance and obliterate the left maxillary sinus, elevate the floor of the orbit and extend into the alveolar bone. During follow-up the lesions were found to grow rapidly, causing pain and swelling

**Fig. 3:**
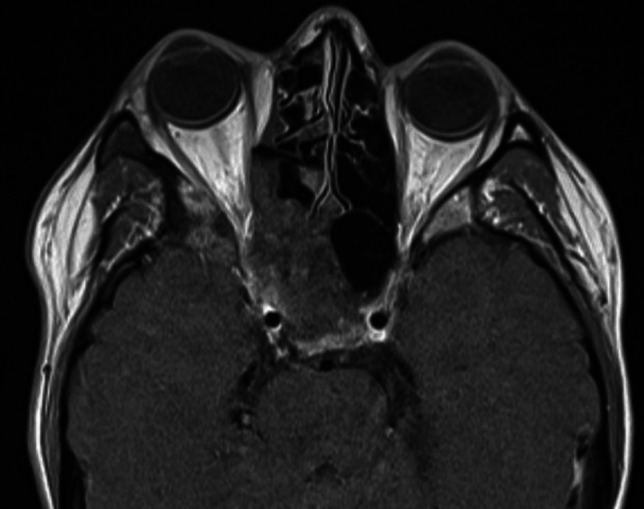
An axial head MRI slice of a 28-year-old female patient with monostotic FD. The image shows a large lesion involving the right frontal bone, ethmoidal bone, maxilla, and the zygomatic bone with subsequent narrowing of the orbit and protrusion of the eye. The patient’s symptoms were facial asymmetry, numbness, feeling of pressure, exophthalmos and ptosis of the right eye and intermittent diplopia

Overall 70 patients (95%) had monostotic FD (MFD) and four (5%) polyostotic FD (PFD). Distribution of lesions per anatomic site is shown in Fig. 1—mandibula and maxilla being the most common ones. The disease caused symptoms in 49 patients (66%) (Figs. 1, 2 and 3). Of the 22 patients diagnosed in childhood, 18 (82%) were symptomatic, whereas only 29 patients (59%) diagnosed in adulthood were symptomatic. Two pediatric and one adult patient had no record of possible symptoms.

In the 49 symptomatic patients, extra-skeletal symptoms occurred in 24 (49%) patients; 10 (42%) suffered from visual symptoms, six (25%) had paresthesia, four (17%) had migraines, three (13%) had hearing loss and one (4%) had trigeminal pain. Chronic pain was recorded in five (21%) and neurological symptoms in 19 (79%) patients. The neurological symptoms included visual defects, such as reduced visual acuity, visual field defects, blurring, obscuration and diplopia, and symptoms of paresthesia, such as complete or partial numbness, and tingling. Others listed included migraine, hearing loss and trigeminal pain. Altogether, the most common symptoms were pain (39%), lesion growth (24%), facial asymmetry (18%), visual symptoms (14%), swelling (12%), paresthesia (8%) and feeling of pressure (7%). All patients with PFD had symptoms. No fractures or malignancies were recorded.

### Diagnostics—radiology and histopathology

A total of 22 (30%) patients were diagnosed in childhood (age < 16 years); for two pediatric patients the exact age was not recorded. The remaining 49 patients (66%) were diagnosed only in adulthood. For three patients the age at diagnosis was not recorded. Mean age of diagnosis was 32.6 years (range 1–79 years); no significant difference was observed between males and females (31.6 vs 33.9 years, respectively).

In total 39 (53%) patients were diagnosed incidentally based on radiologic findings. Incidental findings were more common in adults than in children (57% vs 50%, respectively, *p* = 0.58) and in females than in males (56% vs 50%, respectively, *p* = 0.12). Radiologic imaging was used as a diagnostic method for all patients, typically magnetic resonance imaging (MRI), computed tomography (CT), orthopantomography (OPG), cone beam computed tomography (CBCT), and less commonly bone scintigraphy, bone densitometry, lateral- and anteroposterior images of the skull, ultrasound, and X-ray of the paranasal sinuses.

Lesion biopsies were obtained from 30 patients (41%). Biopsy was performed more commonly in childhood (59%) than in adulthood (33%) and almost equally often in both genders (females 44% and males 38%). There was no information regarding biopsies for four patients.

### Treatment strategies, outcomes, and complications

Altogether 54 patients (73%) were managed by observation only (Table 1), consisting of regular clinical follow-ups, radiographic imaging, and occasionally laboratory testing. Possible expansion and changes in appearance, indicating disease activity, were followed up with imaging. Laboratory tests included bone markers, such as alkaline phosphatase (ALP), which rise during lesion activity. Of these 54 patients, 20 (37%) developed new symptoms during follow-up without prompting further treatment. These included occasional pain or sensations, lesion growth, bone prominence or facial asymmetry, tingling, complete or partial numbness, visual symptoms such as reduced visual acuity, visual obscuration, or difficulty to open eye, feeling of pressure, changes in occlusion or malocclusion, increased swelling, reduced hearing, headache, and nasal congestion. Eight patients (15%) had lesion growth.

**Table 1:** Disease management and treatment complications in 74 Finnish patients with fibrous dysplasia of the head and neck

Disease management	N (x)	% of total N
Observation only	54	73
Given treatment	20 (8)	27
Bisphosphonate treatment	10 (4*)	50
Surgical treatment	6 (5**)	30
Bisphosphonates and surgery	4 (1*,**)	20

x number of patients with complications

*Side effects from bisphosphonate infusions

**Complications from the surgical treatment, such as nerve damage, exposure of fixation material, postoperative thrombosis, and facial asymmetry

The remaining 20 patients (27%) received treatment; ten bisphosphonate treatment, six surgical treatment and four both (Table 1). Mean age at beginning of treatment was 24.3 years (range 9–62 years). Mean age of patients receiving both bisphosphonates and surgery was slightly lower than those receiving only bisphosphonates or surgery (18.5 vs 22.9 and 26.7 years, respectively). Indications for bisphosphonate treatment were lesion expansion during follow-up, headache or other pain, elevated bone markers indicating disease activity, lesion location close to the optic canal, and swelling. Used bisphosphonates included zoledronic acid and pamidronate. In two cases, surgery was considered only if urgent or necessary, such as optic compression. In addition, following indications were reported: narrowing of the ear canal, tenderness, and desire to remove a tumorous or exostotic lesion. Four patients were operated for cosmetic issues.

Of the 14 patients treated with bisphosphonates ten (71%) had no complications (Table 1). Three patients (21%) experienced alleviation of pain, and one patient reduced facial swelling and improved facial symmetry. In two patients (14%) treatment slowed lesion growth and in three (21%) stopped growth completely. One patient received bisphosphonate treatment for a year without any response. Of those who underwent surgery, five (50%) had a good response to treatment without complications. For two (20%), the procedure was done for diagnostic purposes and for two as corrective operations to achieve better facial symmetry.

## Discussion

Fibrous dysplasia (FD) is a rare bone disease characterized by lesions with aberrant bone maturation and replacement with fibrous tissue. While the disease course is usually benign, lesions affecting craniofacial structures can result in a spectrum of symptoms ranging from milder, such as pain and facial deformity, to more severe, such as hearing or vision loss. As patients with FD of the head and neck are scarce, the right diagnostic and treatment algorithms remain incompletely defined. This study describes clinical findings and line of management in altogether 74 patients with FD of the head and neck.

The patient demographics were similar to previous reports with an equal gender distribution and diagnosis usually in early adulthood (mean age 32.6 years) [2, 12, 13]. However, the age at diagnosis varied greatly; symptomatic lesions were usually diagnosed in childhood whereas in adults the diagnosis was often based on an incidental finding, congruent to earlier reports [3, 14, 15]. Interestingly, age at diagnosis was slightly higher for females (33.9 vs 31.6 years), which could be due to men being imaged more frequently for other reasons such as trauma. Monostotic FD was significantly more common (95% of the patients), which is higher than in prior reports. Cheng et al. reported MFD in up to 70% of their craniomaxillofacial FD patients [2] while a more recent meta-analysis by Yang et al. reported no such difference in prevalence of MFD vs PFD [16]. The differences in numbers could be due to variations in diagnostic criteria and definition of a monostotic lesion. In our case, contiguous lesions affecting multiple anatomic sites were considered monostotic. The anatomic distribution of lesions did not differ with literature as mandibula, maxilla and sphenoid bone were the most common sites (Fig. 1; [16]).

Majority (66%) of patients had symptoms, with pain, lesion growth and facial asymmetry being the most common. Although previous reports often describe craniofacial lesions to present as painless, slowly growing lumps, deformities or unilateral swellings [3], pain and facial asymmetry are well-recognized symptoms of FD and often prompt patients to seek treatment [1]. FD-related pain is often described as a headache or another sensation [17], possibly leading to mis- or delayed diagnosis. None had malignant transformation, which is rare (1–4%; [18–20]) and more frequent in PFD, MAS and Mazabraud syndrome.

For all patients, diagnosis was primarily based on imaging findings. FD lesions typically have a peculiar ground-glass appearance on radiographic images and imaging findings are generally considered to suffice a diagnosis [3]. Initial imaging modalities often include CT and OPTG, MRI can be used to further characterize the shape of the lesion, possible extension to adjacent soft tissues and malignant transformation [1, 21] and skeletal gamma imaging to determine disease extent [1]. If necessary, histopathology can be used to confirm diagnosis, as was done for 41% of our patients. There are specific histologic patterns for FD, the Chinese character form composed of irregularly shaped bony trabeculae being the classic one for lesions in limb bones and axial skeleton, whereas the Pagetoid pattern with dense, sclerotic trabecular bone is often observed in craniofacial lesions and the hypercellular pattern composed of discontinuous, often parallelly distributed bone trabeculae in gnathic bone lesions [22]. Lastly, though rarely used and for none in our cohort, patients with syndromic features (suggesting MAS) may undergo genetic testing. Bone-related laboratory testing, especially alkaline phosphatase (ALP) levels, have been mainly used in follow-up [23, 24]; elevated ALP levels may indicate lesion activity and growth tendency while declining levels have been shown to indicate response to bisphosphonates [2, 23, 24]. It has also been reported that an increase in ALP levels combined with aggressive behavior of the lesion could indicate malignancy [25].

As no curative treatment exists, the main treatment goals for craniofacial FD are to improve functional capacity, alleviate pain, and attain satisfactory cosmesis. Treatment is individually tailored depending on patient age and the location, growth, and symptoms of the lesion [3, 26]. Majority (73%) of our patients were chosen for observation (Table 1), as recommended for patients with stable disease [1, 27]. However, others have reported a more active approach. Thompson et al. conducted a study with 26 FD patients, of which only two patients were opted for observation [28] and Bertin et al. reported a cohort of 12 patients with FD of the orbital region of whom three patients received no treatment [29].

For symptomatic and progressive lesions, bisphosphonates were most commonly used (70% of those treated). Intravenous bisphosphonates are the mainstay medication, although data on their efficacy is conflicting [3]. By reducing bone resorption, they have been shown to slow craniofacial lesion growth and alleviate pain [30]. Of those treated, 21% experienced alleviation of pain, one patient reduced facial swelling and better facial symmetry, for 14% the treatment slowed lesion growth and for 21% lesion growth stopped completely. Chapurlat et al. reported complete clinical response to pamidronate in 8 of 13 patients with bone pain and partial response in the remaining 5 patients. Radiologic changes (progressive filling of osteolytic areas and cortical thickening) were observed in 9 of 20 patients. Serum ALP and urinary type I collagen C-telopeptide levels decreased significantly during treatment [31]. Similarly, Majoor et al. showed complete response in 24/30 of their PFD patients, while only 4/11 of MAS patients seemed to benefit from treatment. Conversely, Plotkin et al. found no radiographic evidence of filling of lytic lesions or cortical thickening in their 18 pediatric and adolescent PFD patients receiving pamidronate [32], and Thomsen et al. reported only 5/23 FD patients with clear decrease in bone pain or radiologic regression of the disease [28].

Of note, 29% of the treated patients had side effects from the infusions. The disease and lesions in FD patients behave differently, which could explain variable drug response. While a high skeletal burden score is seen as a significant risk factor for incomplete response to bisphosphonate therapy among PFD/MAS patients [33], no clear correlation has been found between bone markers, disease type (MFD or PFD), age at onset of treatment, and serum phosphate levels and radiological response [34]. Recent clinical studies have shown promising results on denosumab, novel monoclonal antibody with inhibiting activity on RANKL, an important ligand driving osteoclastogenesis [35]. Denosumab has been found to alleviate pain and restrain the growth and formation of new lesions [36, 37]. However, discontinuation of the drug is associated with a rebound in bone turnover and hypercalcemia [38].

A third of our cohort underwent surgery with generally good outcomes. Surgery was typically given as second-line treatment and patients were slightly older compared to those receiving bisphosphonates (26.7 vs 22.9 years of age respectively). Indications for surgery were aesthetic issues, location in the orbita, narrowing of the ear canal, tenderness to touch, and a tumor-like or exostotic lesion that was wanted to be removed. These are congruent to earlier findings with facial asymmetry being the most common [39]. The extent and type of surgery is tailored individually according to lesion location, secondary complications and associated symptoms and warrants careful planning. Although the outcomes are generally good and beneficial to patient quality of life, possible complications include postoperative growth of the lesion, changes in facial functions such as speech and chewing, and nerve damage [39, 40]. In our cohort, 50% of treated patients had complications with nerve damage as the most common complication.

We recognize certain limitations to our study, mainly concerning the relatively small cohort size and the study's retrospective nature. These may introduce selection bias and give a limited view of the true disease course, scope of symptoms, disease management and diagnostic and treatment outcome. Given the high incidence of incidental diagnoses, milder forms of FD may go unrecognized and give a distorted image on disease severity and need for active treatment. However, considering the often stagnant or slowly progressive disease course as well as the great rarity of the disease, a prospective study would require an extremely long follow-up period to obtain a representative patient population. Therefore, and regardless of these limitations, we consider our study to offer unique and recent insight into patients with head and neck FD and to provide a valuable contribution to the literature. Focus on pediatric patients with active disease and prospective approach could potentially provide deeper understanding of the disease course and its management in the future. Use of genetics in diagnostics is becoming more common and may open new possibilities in diagnostics and management.

In conclusion, patients with FD of the head and neck are a very heterogeneous group and the disease manifests in a very variable manner depending on the number, anatomic location and the extent of the lesions. Thus, individualized management is warranted. Most diagnoses are incidental and can often be managed by observation only. Despite this, craniofacial lesions impose possible risks to adjacent, often vital structures and may require more careful follow-up and more active treatment approach. Future studies are recommended to better define the disease characteristics and hopefully offer new avenues for targeted treatment.

## Data Availability

Data is available upon request from corresponding author.
